# Interaction of graphene family materials with *Listeria monocytogenes* and *Salmonella enterica*

**DOI:** 10.1186/s11671-015-0749-y

**Published:** 2015-01-28

**Authors:** Natalia Kurantowicz, Ewa Sawosz, Sławomir Jaworski, Marta Kutwin, Barbara Strojny, Mateusz Wierzbicki, Jacek Szeliga, Anna Hotowy, Ludwika Lipińska, Rafał Koziński, Joanna Jagiełło, André Chwalibog

**Affiliations:** Department of Animal Nutrition and Biotechnology, Faculty of Animal Science, Warsaw University of Life Sciences, Ciszewskiego 8, 02-786 Warsaw, Poland; Institute of Electronic Materials Technology, Wólczyńska 133, 01-919 Warsaw, Poland; Department of Veterinary Clinical and Animal Sciences, University of Copenhagen, Groennegaardsvej 3, 1870 Frdereiksberg C, Copenhagen, Denmark

**Keywords:** Pristine graphene, Graphene oxide, Reduced graphene oxide, *Listeria monocytogenes*, *Salmonella enterica*, Bacteria growth

## Abstract

Graphene family materials have unique properties, which make them valuable for a range of applications. The antibacterial properties of graphene have been reported; however, findings have been contradictory. This study reports on the antimicrobial proprieties of three different graphene materials (pristine graphene (pG), graphene oxide (GO), and reduced graphene oxide (rGO)) against the food-borne bacterial pathogens *Listeria monocytogenes* and *Salmonella enterica*. A high concentration (250 μg/mL) of all the analyzed graphenes completely inhibited the growth of both pathogens, despite their difference in bacterial cell wall structure. At a lower concentration (25 μg/mL), similar effects were only observed with GO, as growth inhibition decreased with pG and rGO at the lower concentration. Interaction of the nanoparticles with the pathogenic bacteria was found to differ depending on the form of graphene. Microscopic imaging demonstrated that bacteria were arranged at the edges of pG and rGO, while with GO, they adhered to the nanoparticle surface. GO was found to have the highest antibacterial activity.

## Background

Due to the development of antibiotic-resistant bacterial strains, there is an increasing need to evaluate and develop alternative methods for antibacterial treatment [1-5]. It has been reported that carbon (i.e., nanotubes and fullerenes) and diamond nanoparticles possess antimicrobial properties [6,7]. Recently, it has also been demonstrated that a new allotrope of carbon, graphene, has antibacterial activity [8]. This activity has also been reported to be more effective than some currently used therapeutic antibiotics [5].

Graphene is a two-dimensional monolayer of carbon atoms which are tightly packed into a flat hexagonal structure, similar to that of a honeycomb lattice [9]. Graphene is regarded as the thinnest material in the world as it is only one carbon atom thick [10], although its surface area may be up to 1 cm^2^ [11]. The ratio of its thickness to surface area is exceptional when compared to other nanoparticles. Moreover, graphene is considered to be an elementary building block for all sp^2^-hybridized carbon allotropes [12]. Defect-free pristine graphene (pG) does not have any dangling bonds on its surface [13]. In contrast, the edges of pG consist of a line of atoms with dangling bonds, differing from the surface in terms of electronic, chemical, and magnetic properties. These unstable dangling bonds are subjected to chemical functionalization under ambient conditions [14]. As a consequence, the nature of the interactions of biological molecules and/or cells with pG is likely to depend on the site of interaction: the surface or edges. Previous findings indicated that glioblastoma cells had a strong affinity for, and adhered to, the surface of pG flakes, rather than the edges [15].

pG however differs both physically and chemically from graphene oxide (GO) and reduced graphene oxide (rGO). pG is manufactured by the exfoliation of graphite, whereas GO is obtained by the oxidation of graphite in the presence of strong acids and oxidants. Subsequent reduction of GO is used to generate rGO [16]. GO differs significantly from other graphene family materials (GFM) due to the disruption of its sp^2^ bonding network. GO also possesses oxygen as a significant chemical component (approximately 30% (*w*/*v*)) in the form of oxide functional groups, which can be mainly classed as either alcohols or epoxides [17]. This results in GO having partial hydrophilic properties, unlike pG [8,18]. rGO is quite different chemically from its GO precursor, instead being more similar to pG [17] due to its hydrophobic π-bond graphene domains [8,18].

GFM have high thermal stability and mechanical strength, with relatively good biocompatibility with humans. These features make them very robust, useful, and multifunctional materials, particularly in light of the increasing evidence of their antibacterial properties [5]. Hu et al. [19] observed that GO had a detrimental effect on *Escherichia coli*, due to decreased bacterial production of ATP. Reduction of GO to rGO, however, resulted in slightly lower antibacterial activity relative to GO, as well as significantly increased the cytotoxicity. Liu et al. [20] explained the antibacterial effect of GO against *E. coli* by the induction of oxidative stress. However, it has also been demonstrated that GO had no detrimental effects on *E. coli* [21]. The effects of GFM on some other types of bacteria have also been reported. GO nanowalls reduced the viability of *Staphylococcus aureus*, as did rGO to a lesser extent [22]. Other studies indicated lack of toxic effects of GO and rGO on *Shewanella* [23]. Yet, the number of studies on the antibacterial activity of pG, GO, and rGO is limited and mechanisms of toxicity or lack of toxicity are not fully explained.

In our previous studies, we examined how interactions of *Salmonella enterica* and *Listeria monocytogenes* with various nanoparticles (diamond, silver, gold, and platinum) affected bacterial morphology [7,24]. In this work, we examined how three different graphene nanostructures affect the chosen food-borne bacteria strains: the Gram-positive (G+) *L. monocytogenes* and Gram-negative (G−) *S. enterica*. The chosen bacteria are pathogenic and morphologically different. The nature of the cell wall is the key difference between G+ and G− bacteria. In general, G+ bacteria have a thick peptidoglycan layer outside cells, while G− strains have a much thinner peptidoglycan layer between their inner and outer membranes.

*L. monocytogenes* is a human bacterial pathogen which causes listeriosis [25], and its sources of infection are mainly associated with raw food and working surfaces in food-processing plants [26]. According to the WHO report from 2008 [27], the worldwide prevalence of listeriosis is up to one case per 100,000 population and mainly affects newborns. The case fatality ratio can be up to 30% whereas in patients without adequate treatment, it can be much higher (up to 70%) [27]. Listeriosis is currently treated by antibiotic therapy (mainly penicillin or ampicillin) or bacteriophages [28,29].

*S. enterica* is also a significant factor of food-associated illness, causing diarrhea in infected individuals, although antibiotic therapy is not usually required. Infection with this bacterium is mainly associated with the consumption of products containing undercooked, or raw, eggs [30]. According to the WHO report from 2008 [27], the worldwide prevalence is up to 100 cases per 100,000 population, although the case fatality ratio is below 1% in industrialized countries.

Diversity of the wall structure between G+ and G− bacteria consequently determines properties of the surface, in which bacteria are exposed to the environment. Moreover, chemistry of the surface of GFM also varies, influencing their interactions with bacteria. In this study, we have compared the antibacterial activity of different forms of GFM towards G+ (*L. monocytogenes*) and G− (*S. enterica*) with special emphasis on the visualization of their interactions.

## Methods

### GFM production and characterization

pG was produced by liquid-phase exfoliation of natural graphite (purchased from Skyspring Nanomaterials, Huston, TX, USA). The purity of the material generated was >99.5%, and it had a specific surface area of 120 to 150 m^2^/g.

GO was prepared by a modified Hummers method using natural graphite flakes (purchased from Asbury Carbons, Asbury, NJ, USA). Graphite (5 g) was added into 125 mL of H_2_SO_4_ containing 3.25 g of KNO_3_, and the mixture was then stirred with a mechanical stirrer for 1 h with a speed of 150 rpm. The mixture was then cooled by transferring it into a water/ice bath where its temperature was kept below 5°C, and KMnO_4_ (15 g) was then gradually added. The resultant reaction mixture was taken out of the water/ice bath and kept at 30°C to 35°C with continuous stirring for 1 h with a speed of 100 rpm. The reaction mixture was then left at room temperature for 14 h without stirring. In the next step, deionized water was added to the stirred mixture (200 rpm) so that its temperature did not exceed 35°C. The reaction mixture was then put into a 35°C water bath and mechanically stirred with a speed of 200 rpm for 1 h. The constantly stirred reaction mixture was then heated to 95°C for 15 min. To stop the reaction, 280 mL of deionized water and 5 mL of H_2_O_2_ were added. The purification process was carried out in two steps. Firstly, the mixture was diluted with a 5% HCl water solution and centrifuged (6,000 rpm, 1 h, 50-mL containers), and the precipitate was separated from the clear supernatant by decantation for removal of sulfate and manganese ions. Secondly, the mixture was diluted with deionized water and centrifuged (6,000 rpm, 1 h, 100-mL containers), and the precipitate was separated from the clear supernatant by decantation. The rinsing with deionized water was carried out four times.

To prepare the rGO, a water suspension of 50 mg of GO was acidified to pH = 1 and heated to 90°C. Then 12 mL of reducing mixture (0.01 g of ammonium iodide, 9 g of hydrated sodium hypophosphite, and 1.21 g of sodium sulfite dissolved in 100 mL of deionized water) was added. A black material (rGO) immediately precipitated. The product was filtered, washed with deionized water, and dried.

The pG, GO, and rGO powders were used to make aqueous suspensions for analysis and/or use in experiments. This was done by adding the required amount of powder to ultrapure water and sonicating the solution at 550 W/m^2^ for 1 h.

### FTIR analysis

The Fourier transform infrared (FTIR) spectra of pG, GO, and rGO were determined with a Vertex 80v (Bruker BioSpin Corporation, Billerica, MA, USA) in the range 500 to 4,000 cm^−1^ using attenuated total reflectance spectroscopy with crystal germanium.

### Bacterial cultivation and preparation

*S. enterica* subspecies *enterica* serovar Enteritidis (ATCC 13076) and *L. monocytogenes* (ATCC 19111) were obtained from LGC Standards (Lomianki, Poland). The strains were stored as spore suspensions in 20% (*v*/*v*) glycerol at −20°C. Prior to their use in experiments, the strains were thawed and the glycerol was removed by washing the bacterial cells with distilled water. The bacteria were then grown on nutrient media: tryptic soy agar (TSA) for *S. enterica* and brain heart agar (BHA) for *L. monocytogenes* (Merck Millipore, Darmstadt, Germany). Sterilization of media was carried out at 121°C for 30 min (Tuttnauer 2450EL, Tuttnauer Ltd., Jerusalem, Israel). The bacteria grown on agar plates were harvested by gently washing them off the agar plates with sterile distilled water. The bacterial suspensions were then centrifuged at 4,000 rpm for 5 min using an Eppendorf MiniSpin centrifuge (Eppendorf, Hamburg, Germany) to pelletize the cells. The bacterial cell pellet was then re-suspended in sterile distilled water.

To calculate the number of bacteria in the cell suspension, the optical density of the suspensions at 600 nm (OD600) was measured using a spectrophotometer (Helios Epsilon, Unicam, Milwaukee, WI, USA). The OD600 readings were then converted to cell numbers using calibration curves. Calibration curves for each bacterium were prepared as follows. Serial tenfold dilutions (up to 10^−5^) of bacterial suspensions of known optical density were performed: 1 mL of each dilution spread on petri dishes containing the nutrient medium. After 24 h of incubation at 37°C, the number of colonies formed on the petri dishes was enumerated. Based on the results of the enumerations (conducted in triplicate), the density of the original bacterial suspension in colony forming units (cfu)/mL was calculated. With both strains, an OD600 reading of ≈ 0.130 was found to correspond to ≈ 3 × 10^8^ cfu/mL.

### Growth inhibition test

Based on the bacterial suspension cell density (determined as outlined above), bacterial suspensions containing ≈ 5 × 10^8^ cfu/mL in 0.85% (*w*/*v*) NaCl were prepared. Aqueous suspensions of pG, GO, and rGO were prepared (as outlined above) at both 25 and 250 μg/mL concentrations. The suspensions were then gently mixed with *S. enterica* or *L. monocytogenes* and incubated overnight (18 h) at room temperature. Control samples of bacteria were treated with ultrapure water. After incubation, serial tenfold dilutions (up to 10^−3^) were prepared. One milliliter of each dilution was transferred to petri dishes with the nutrient medium (TSA for *S. enterica* and BHA for *L. monocytogenes*), and after 24 h of incubation at 37°C, the number of colonies formed was enumerated. All incubations were conducted in triplicate. Based on the results of the plate counts, the number of live bacteria was determined in each of the samples and controls.

### Visualization of GFM and their interaction with bacteria

The shape of the GFM was inspected by a digital camera, scanning electron microscope (SEM), and transmission electron microscope (TEM). The macroscopic structure of GFM powder was visualized using the digital camera Nikon D7000 with the lens Nikon AF-S Micro-Nikkor 105 mm f/2.8G IF-ED VR (Nikon, Tokyo, Japan). SEM analysis of the GFM was performed by means of an FEI Quanta 200 electron microscope (FEI Co., Hillsboro, OR, USA). All imaging was performed in triplicate. Samples of GFM aqueous suspensions (25 μg/mL) for TEM observations were prepared by placing droplets of the suspension onto formvar-coated copper grids (Agar Scientific Ltd., Stansted, UK). Immediately after the droplets had air-dried, the grids were inserted into the TEM for observation with the JEM-2000EX TEM at 80 keV (JEOL, Tokyo, Japan), and images were captured with a Morada 11 megapixel camera (Olympus Soft Imaging Solutions GmbH, Münster, Germany).

Samples for TEM visualization of the interaction of the GFM with each bacterium were prepared by mixing suspensions (200 μL of 25 μg/mL) of pG, GO, and rGO with bacterial cell suspensions (200 μL containing ≈ 5 × 10^8^ cfu/mL in 0.85% NaCl). Control samples of bacteria were treated with ultrapure water. The samples were gently mixed for 15 min at room temperature, and then droplets of the samples were placed onto formvar-coated copper grids and observed by TEM.

### Size distribution and zeta potential measurements

The zeta potential and size distribution of the GFM were measured using a dynamic laser scattering method. The GFM (pG, GO, and rGO) were suspended in ultrapure water and measured on a Zetasizer Nano-ZS90 (Malvern Instruments Ltd., Malvern, UK). Each sample (25 μg/mL) was measured after 120 s of stabilization at 25°C in four replicates.

The zeta potential was measured separately for each bacterial strain before and after application of the GFM. Suspensions of pG, GO, and rGO in ultrapure water (200 μL of 25 μg/mL) were added to bacterial cell suspensions (200 μL containing ≈ 5 × 10^8^ cfu/mL in 0.85% NaCl) and incubated for 15 min at 37°C. Control samples of bacteria were treated with ultrapure water.

### DPPH test

To measure the prooxidant effect of the GFM, a 2,2-diphenyl-1-picrylhydrazyl (DPPH) assay was performed. Prooxidant activity was also measured by using the modified DPPH method [13]. To estimate the prooxidant effect of the GFM, the DPPH radical was first reduced by ascorbic acid (Merck, Darmstadt, Germany) with various concentrations (10 mg/L, 1 mg/L, 100 μg/L, 10 μg/L, and 1 μg/L) and optimized to the concentration of 100 μg/L. After reduction, the oxidizing properties of the GFM were observed as an increasing amount of free DPPH radical that was generated. To examine the concentration effect of the GFM (25, 50, 100, and 250 μg/mL), 10 μL of each GFM aqueous suspension was mixed with 190 μL of DPPH solution. The samples were vortexed and allowed to scavenge DPPH in the dark for 30 min. The absorbance of the reaction mixture was measured at 517 nm in a spectrophotometer (Infinite M200 Pro, Tecan, Männedorf, Switzerland). In all the cases, measurements were done in triplicate. The scavenging percentage was calculated using the formula:

$$ \mathrm{DPPH}\kern0.5em \mathrm{scavenging}=\frac{\left({A}_{\mathrm{C}}-{A}_{\mathrm{S}}\right)\times 100}{A_{\mathrm{C}}} $$

where *A*_C_ and *A*_S_ are the absorptions of blank DPPH and DPPH subjected to interact with the sample at 517 nm, respectively.

### Data analysis

Statistical significance was determined by one-way analysis of variance (ANOVA) using Statgraphics® Plus 4.1 (StatPoint Technologies, Warrenton, VA, USA). Differences at *P* ≤ 0.05 were defined as statistically significant.

## Results

### Physical and chemical characterization

**Figure 1:**
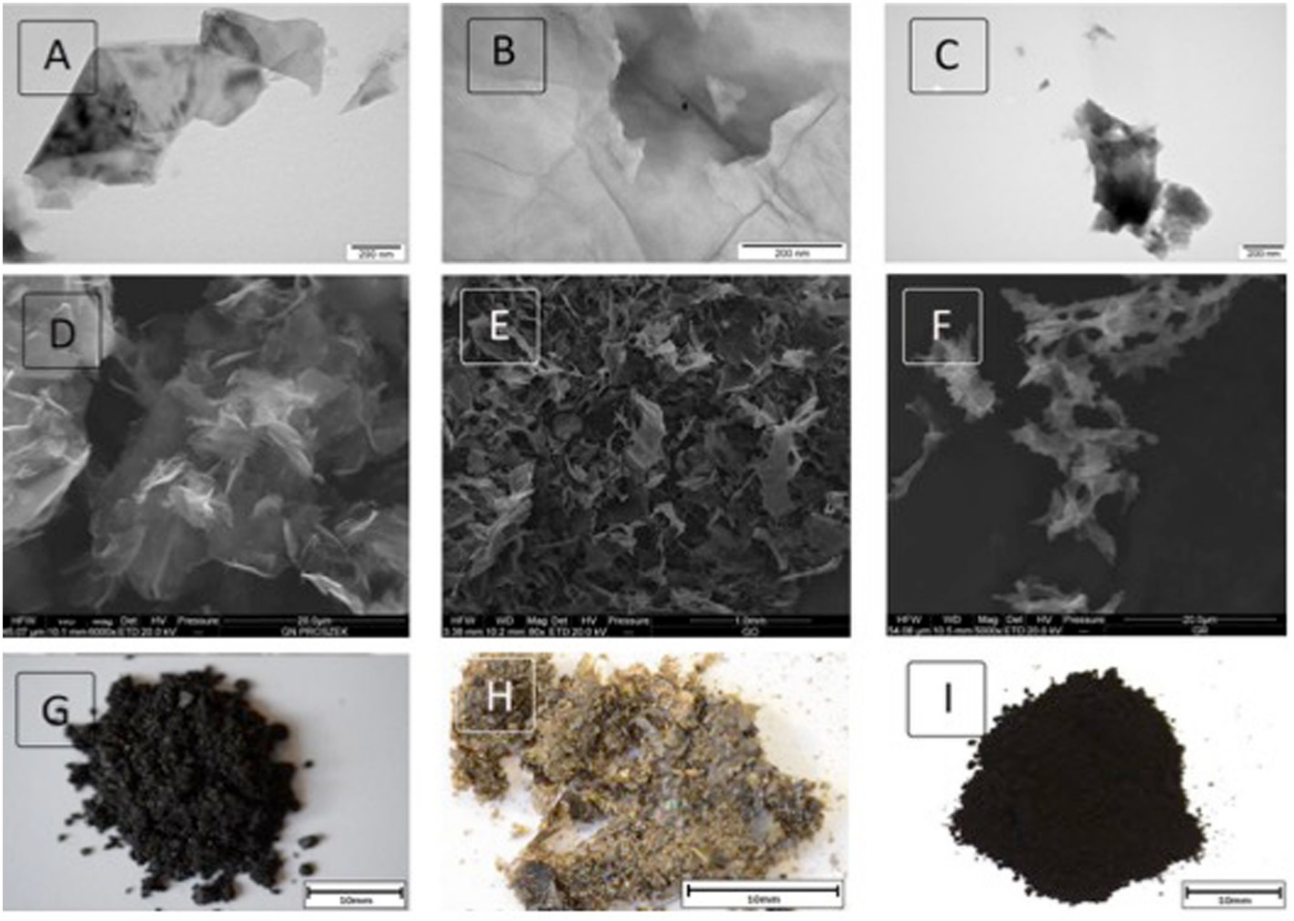
**GFM were visualized using transmission electron microscopy (A-C), scanning electron microscopy (D-F), and a digital camera (G-I).** Images of pristine graphene **(A, D, G)**, graphene oxide **(B, E, H)**, and reduced graphene oxide **(C, F, I)**.

**Figure 2:**
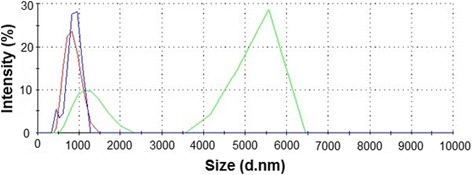
**Size distribution of different graphene family materials, with intensity indicative of their concentration.** Line color coding representative spectra: pristine graphene (blue), graphene oxide (green), and reduced graphene oxide (red). Triplicate measurements.

The shape of the nanoparticles, as visualized by the digital camera, TEM, and SEM, differed between the individual GFM (Figure 1, Table 1). The shape of GO was observed to be mainly a large film-like layer (Figure 1B,E); the powder was light brown (Figure 1H). pG was comprised of flakes that were smaller than the GO film-like form and consisted of one or more layers (Figure 1A,D); the powder was dark (Figure 1G). rGO was also in the form of flakes but contained more layers than pG (Figure 1C,F) and was the darkest (Figure 1I). GO had the smallest size distribution by intensity and the peak was around 1,000 nm; a slightly higher peak was seen for pG. Two peaks, over 1,000 and 5,600 nm, characterized rGO (Figure 2).

**Table 1:** **Summary of the physical and chemical properties of pG, GO, and rGO**

	**pG**	**GO**	**rGO**
Shape	Irregular, angular, single to a few layers	Film-like, rounded, single layers	Irregular, frayed, a few layers
Average size (μm)	1.86 ± 0.6	1.27 ± 0.1	2.53 ± 0.2
Zeta potential (mV)	−17.7 ± 4.3	−49.8 ± 1	−25.1 ± 2.6
Surface chemical bonds	C = C	O-H, C = C, C = O, C-O, C-H	C = O, C = C, C-O

Shape was estimated upon analysis of scanning electron microscopy pictures. Zeta potential and average size were measured by Zetasizer. The content of chemical bonds was identified by Fourier transform infrared spectra analysis.

**Figure 3:**
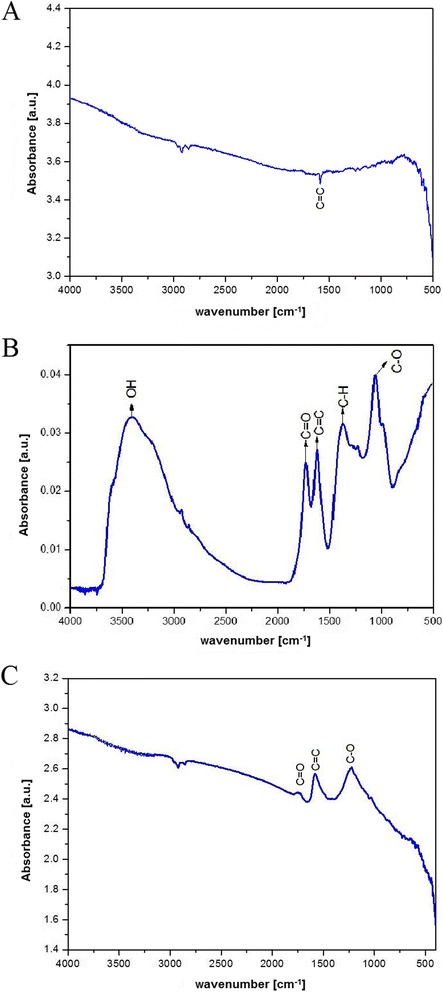
**FTIR spectra of graphene family materials: pristine graphene (A), graphene oxide (B), and reduced graphene oxide (C).**

The chemical structure of the surfaces of the GFM was found to differ greatly (Figure 3). The FTIR spectrum of pG (Figure 3A) indicated the absence of hydroxyl (O-H) groups, with only alkene (C = C) bonds detected. The FTIR spectrum of GO in contrast was much more complex (Figure 3B), indicating the presence of the following bonds: ν_C=O_ (1,731 cm^−1^), ν_C=C_ (1,621 cm^−1^), δ_C-H_ (1,375 cm^−1^), and δ_C-O_ (1,059 cm^−1^). The reduction of GO to rGO resulted in a less complex FTIR spectrum compared to that of GO (Figure 3C). Three peaks at 1,769, 1,602, and 1,289 cm^−1^ were observed, corresponding to carboxylic acid (C = O), alkene (C = C), and ether (C-O) bonds, respectively. No significant absorptions associated with hydroxyl groups were observed in the spectrum, unlike GO where large amounts were detected. The differences between the GO and rGO spectra therefore demonstrated that the reduction process removed both hydroxyl and carboxylic acid functional groups. These findings are consistent with the different hydrophilic properties of the material (Table 1).

### Toxicity

**Figure 4:**
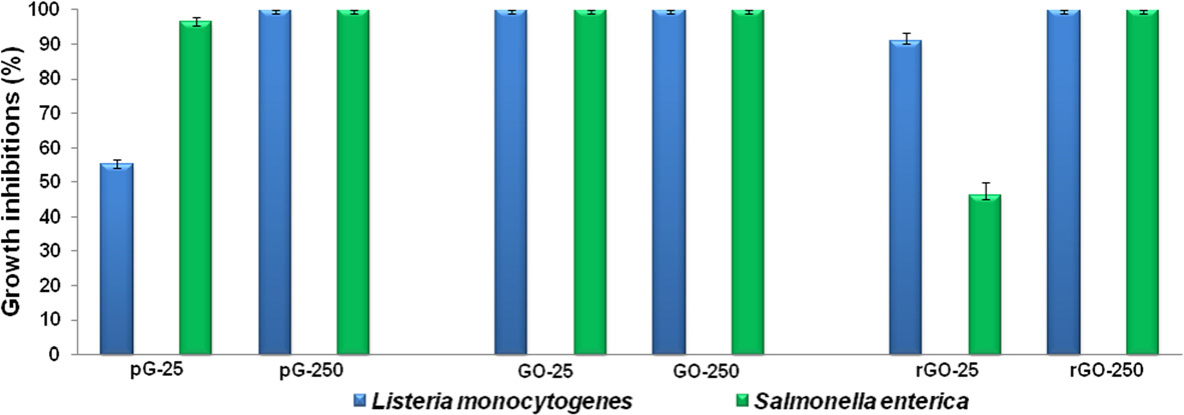
**Influence of pG, GO, and rGO on the growth of** ***Listeria monocytogenes*** **and** ***Salmonella enterica*** **at 25 and 250 μg/mL.** Data presented are the average of triplicate determinations, with error bars representing mean standard error.

The high concentration (250 μg/mL) of pG, GO, and rGO consistently inhibited the growth of *S. enterica* and *L. monocytogenes* by 100% (Figure 4). At a lower concentration (25 μg/mL), only GO totally inhibited the growth of both bacteria, by 100% and 99.9%, respectively. pG inhibited the growth of *S. enterica* (96.5%) more than *L. monocytogenes* (54.5%), while rGO inhibited the growth of *L. monocytogenes* (91%) more than *S. enterica* (46%).

### Interactions with bacteria

**Figure 5:**
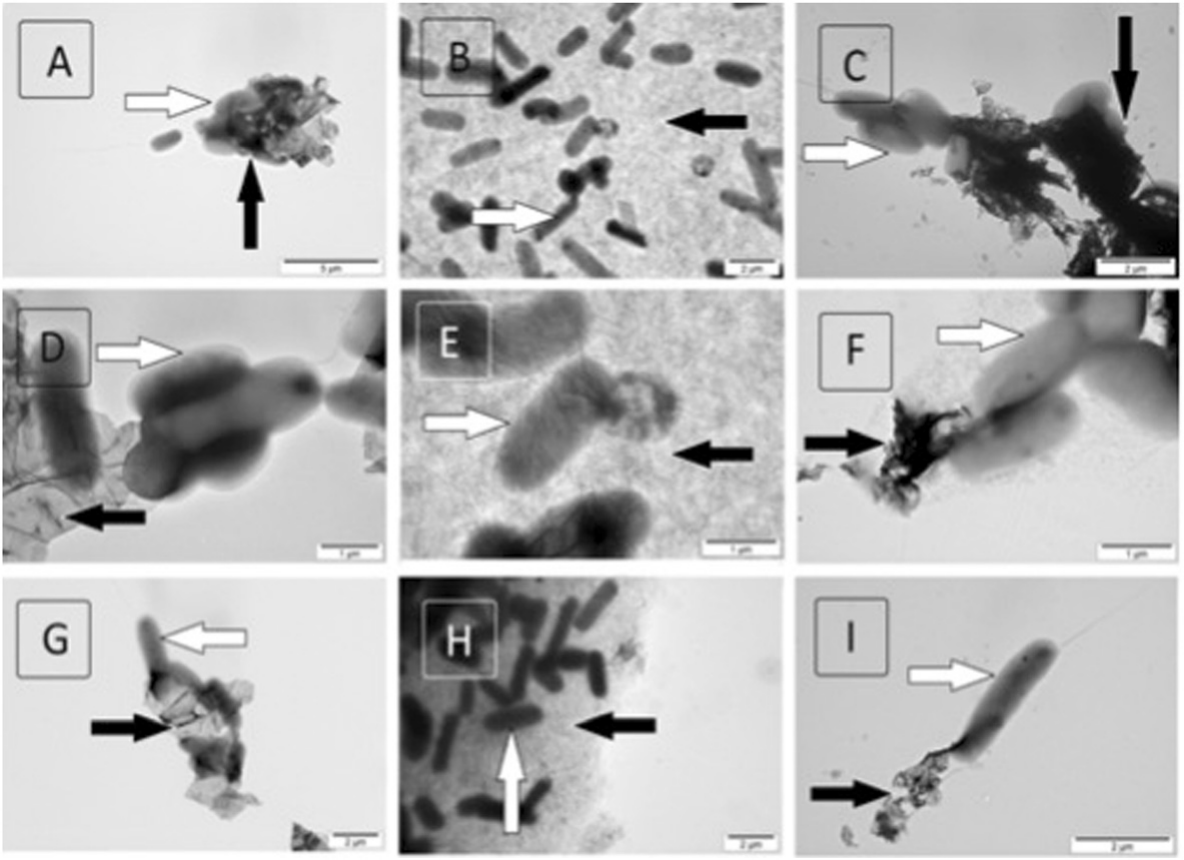
**Visualization of the interaction of graphene family materials with** ***Salmonella enterica*** **using transmission electron microscopy.** Pristine graphene **(A, D, G)**, graphene oxide **(B, E, H)**, and reduced graphene oxide **(C, F, I)**. Black arrows indicate the graphene material and white arrows the bacterial cells.

**Figure 6:**
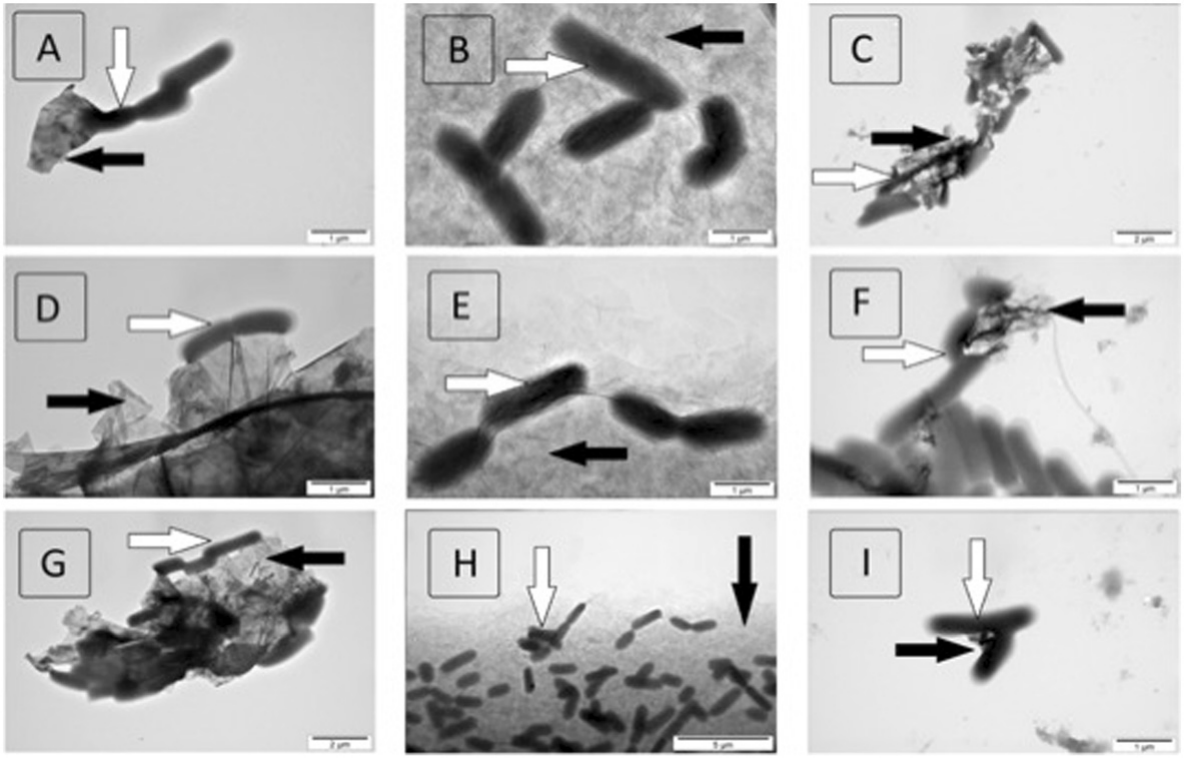
**Visualization of the interaction of graphene family materials with** ***Listeria monocytogenes*** **using transmission electron microscopy.** Pristine graphene **(A, D, G)**, graphene oxide (**B, E, H)**, and reduced graphene oxide **(C, F, I)**. Black arrows indicate the graphene material and white arrows the bacterial cells.

The nanoparticle-bacteria self-organization as a result of the interaction of the GFM and *S. enterica* (Figure 5) and *L. monocytogenes* (Figure 6) was observed. Both bacteria showed a strong affinity and attachment to all the forms of graphene tested; however, the methods of interaction differed between the GFM. The bacteria, which adhered to GO, were distributed over the entire surface of the flakes (Figures 5B,H and 6B,H). It appeared that the bacteria were partially pressed into the GO surface, with wrinkles in the GO layer evident around the adherent bacteria (Figures 5E and 6E). In contrast, the bacteria preferentially attached to the edges of the flakes of pG and rGO surrounding the flakes or forming chains of bacteria that pulled the flakes apart (Figures 5A,C,D,F,G,I and 6A,C,D,F,G,I).

### Zeta potential

**Figure 7:**
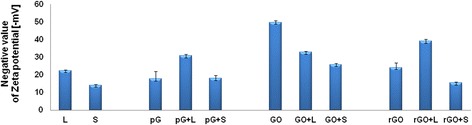
**Effect of 25 μg/mL concentration of pG, GO, and rGO on the zeta potential of** ***L*** **.** ***monocytogenes*** **(L) and** ***S*** **.** ***enterica*** **(S).** Data presented are the average of triplicate determinations, with error bars representing mean standard error.

The zeta potential of the GFM differed, although all of them generated negative values (Table 1). Both bacteria also had negative zeta potential values (Figure 7), with the value for *L. monocytogenes* (−22.2 mV) being lower than that for *S. enterica* (−14.1 mV). Interaction of bacteria and the GFM resulted in the change of zeta potential. *L. monocytogenes* increased the zeta potential of pG and rGO, whereas *S. enterica* decreased the zeta potential of GO and rGO.

### Prooxidative properties

**Figure 8:**
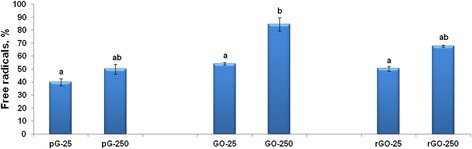
**Effect of pG, GO, and rGO at 25 and 250 μg/mL on the oxidation of the reduced DPPH radicals.** Data presented are the average of triplicate determinations, with error bars representing mean standard error. Values with different superscripts are significantly different (*P* ≤ 0.05).

To observe the prooxidative properties of the GFM, DPPH radicals were reduced with ascorbic acid and then the influence of the GFM on the oxidation of DPPH (resulting in the formation of free DPPH radicals) was assessed. All the GFM oxidized the reduced DPPH at both concentrations (Figure 8). However, only with GO did the 250 μg/mL concentration result in a significant increase in free radical formation compared to the 25 μg/mL concentration.

## Discussion

In the present study, the antibacterial properties of three different forms of graphene were compared. At a high concentration (250 μg/mL), all the GFM totally inhibited the growth of both *S. enterica* and *L. monocytogenes*. A lower concentration (25 μg/mL) of GO also completely decreased the growth of the bacteria. As far as we know, this is the first report of an antimicrobial effect of GO against *L. monocytogenes*. Also, in the case of the G− *Salmonella*, the only one experiment with *Salmonella typhimurium* was carried out. Veerapandian et al. [31] documented that the minimum inhibitory concentration of GO for *S. typhimurium* was 0.25 μg/mL and the minimum bactericidal concentration (MBC) was 0.5 μg/mL. The MBC of GO against other bacteria was also tested: *E. coli* (0.5 μg/mL), *Bacillus subtilis* (1 μg/mL), and *Enterococcus faecalis* (2 μg/mL), respectively. The same tendency was observed by Zhang et al. [32]; however, it was reported that a higher concentration of GO (10 μg/mL) was needed to decrease the growth of *E. coli*.

Bacteriostatic effects of GO, rather than bactericidal, have been reported [21,32]. Bao et al. [33] also showed that only a small zone of bacterial inhibition occurred around GO during disc diffusion tests with *E. coli* and *S. aureus*. In contrast, a recent study [34] with eight different bacterial species showed that GO had no antibacterial effect. In fact, it has even been reported that GO can promote *E. coli* growth [35]. Interestingly, Chen et al. [36] reported that GO promoted the growth of the gut microbe *Bifidobacterium adolescentis* and had an antagonistic effect on the pathogens *E. coli* and *S. aureus*.

These contrasting observations of the antimicrobial properties of GO may be due to the lack of standardization of GO preparations, producing particles with different sizes and numbers of sheets. In addition, differences in the methodologies employed to assess antibacterial activity may also influence the findings of the studies. In order to circumvent some of these issues, a TEM-based approach was used in this study. Imaging data demonstrated that GO had a strong affinity towards *S. enterica* and *L. monocytogenes*. In fact, the affinity was so strong that there were no bacteria visible in the field of view, other than that attached to the GO. Moreover, bacteria were distributed on the surface as individual cells with no colonies evident, as previously observed [20]. That unusual bacteria-GO self-organization could be characterized by binding, available on the GO surface. On the photos from TEM, it was seen that bacterial cells were attached to the GO surface, not rinsed off the nanoparticles during the TEM sample preparation.

Some bacterial species have the ability to reduce GO by electrotransfer, a process mediated by their cytochromes: MtrA, MtrB, and MtrC/OmcA [37]. Therefore, we examined the antioxidant status of the bacteria, GFM, and the interaction resulting from the mixture of both. Neither bacterial species, despite differences in their cell wall characteristics, produced free DPPH radicals (data not shown). There was no significant effect of pG and rGO on the oxidation of reduced DPPH radicals; however, when the reduced DPPH was treated with GO, it was strongly oxidized to form free DPPH radicals, demonstrating the prooxidative property of GO. This property was also observed by Chang et al. [38] in experiments with adenocarcinomic human alveolar basal epithelial A549 cells. Furthermore, Liu et al. [38] concluded that graphenic carbon surfaces react with oxygen to create a surface-bound C(O_2_) intermediate, which oxidizes reduced molecules to their oxidized form. It was also suggested that the oxidation process took place mainly at the edges of the graphenic carbon or at the defective sites on the surface [20]. The present findings, however, clearly indicate that the bacteria were placed only on the surface of GO. Coluci et al. [39] reviewed the molecular structure of GO and reported the presence of highly oxidized polyaromatic carboxylated fragments (oxidative debris) on the surface of GO. It was speculated that bacteria might preferentially interact with these fragments. Oxidative debris would be chemically reduced after the process of forming rGO, resulting in different nanoparticle properties relative to preferred sites of bacterial attachment.

In this study, pG and rGO were found to have a lower antibacterial activity than GO. At a low concentration, the growth of the bacteria was decreased to different extents, whereas at a high concentration, the growth was similarly inhibited. The results are in line with measurements of antibacterial activity using *E. coli* [19,20]. The antibacterial effects of pG and rGO at lower concentrations differed with the G+ *L. monocytogenes* and G− *S. enterica*. The differences could be attributed to different structures of cell walls. The cell wall of G+ bacteria is a thick (20 to 50 nm) peptidoglycan layer, whereas in G− bacteria, a thin (7 to 8 nm) peptidoglycan layer is located between an inner and outer cell wall membrane that is mainly comprised of phospholipids. *S. enterica* therefore has an outer phospholipid membrane, which can directly interact with the hydrophobic domains of both rGO and pG, compromising the integrity of the bacterial cell wall. Nevertheless, despite *L. monocytogenes* having a thick peptidoglycan layer protecting its phospholipid membrane, it was also susceptible to the antibacterial activity of the GFM. As the construction of the bacterial cell wall is very complex, further mechanisms may well be characterized in future studies.

Moreover, in the present study, TEM visualization showed that interactions between the bacteria and pG and rGO were quite different from that of GO. Bacteria were mainly attached to the edges of the pG and rGO flakes, with a specific interface between the cells and the flakes, which appeared as electron dense lines. This was characteristic of interactions with both *S. enterica* and *L. monocytogenes*. Furthermore, the rGO flakes had the appearance of being very rugged and being a potentially harmful structure for bacteria to encounter, in comparison to the smooth film-like form of GO. It was observed that the bacteria had a tendency to form chains on the edges of rGO, in contrast to the single cells distributed over the entire surface of GO. These differential bacterial interactions with the GFM can be explained by their different chemical properties. Reduction of GO dramatically changes the chemistry of its surface. According to Stankovich et al. [40], the X-ray photoelectron spectroscopy spectrum of GO indicates a considerable degree of oxidation with different functional groups: the non-oxygenated ring C, the C in C-O bonds, the carbonyl C, and the carboxylate carbon (O-C = O). rGO also exhibits the same oxygen-containing groups as GO but in a much smaller amount. The surface of GO is partially hydrophobic with hydrophilic regions, uncharged, and with polar groups (i.e., -OH or = O). After the reduction of GO, the rGO formed becomes hydrophobic in nature [18]. This is consistent with the zeta potential findings, as after reduction the partially hydrophobic and well-dispersed GO (−49.8 mV) changes its hydrophilic nature and also becomes less dispersed in colloidal suspension (−25.1 mV). The stability of GFM in aqueous suspension, and consequently the surface area available for bacterial interaction, plays an important role in biological interactions as well as potentially influencing the toxicity of GFM. Despite this, however, it is the affinity of the GFM-exposed chemical groups to biomolecules that primarily determines the nature of the interaction. Reactive functional groups available on GFM edges are still not well recognized, despite there being a considerable amount of carboxyl groups [41], although this point is still a matter of debate [42].

Taking into consideration that the edges of pG and rGO are relatively rich in carboxyl groups, we can hypothesize that these groups may be an attractive site for bacterial attachment. Carboxyl groups are present on a large range of nutritional molecules (i.e., amino acids, short-chain organic acids, and fatty acids), which are commonly recognized, metabolized, and consumed by bacteria. The carboxyl groups available on the pG and rGO edges are therefore speculated to play a ‘baiting’ role for attracting bacteria. Consequently, the antibacterial activity of rGO and pG might be attributed to two different mechanisms. The sharp flake edges may have detrimental effects on the integrity of cell membranes [22]. Also, the chemical affinity of the hydrophobic areas of pG or rGO to phospholipid membranes may lead to the destruction of these structures [18].

**Figure 9:**
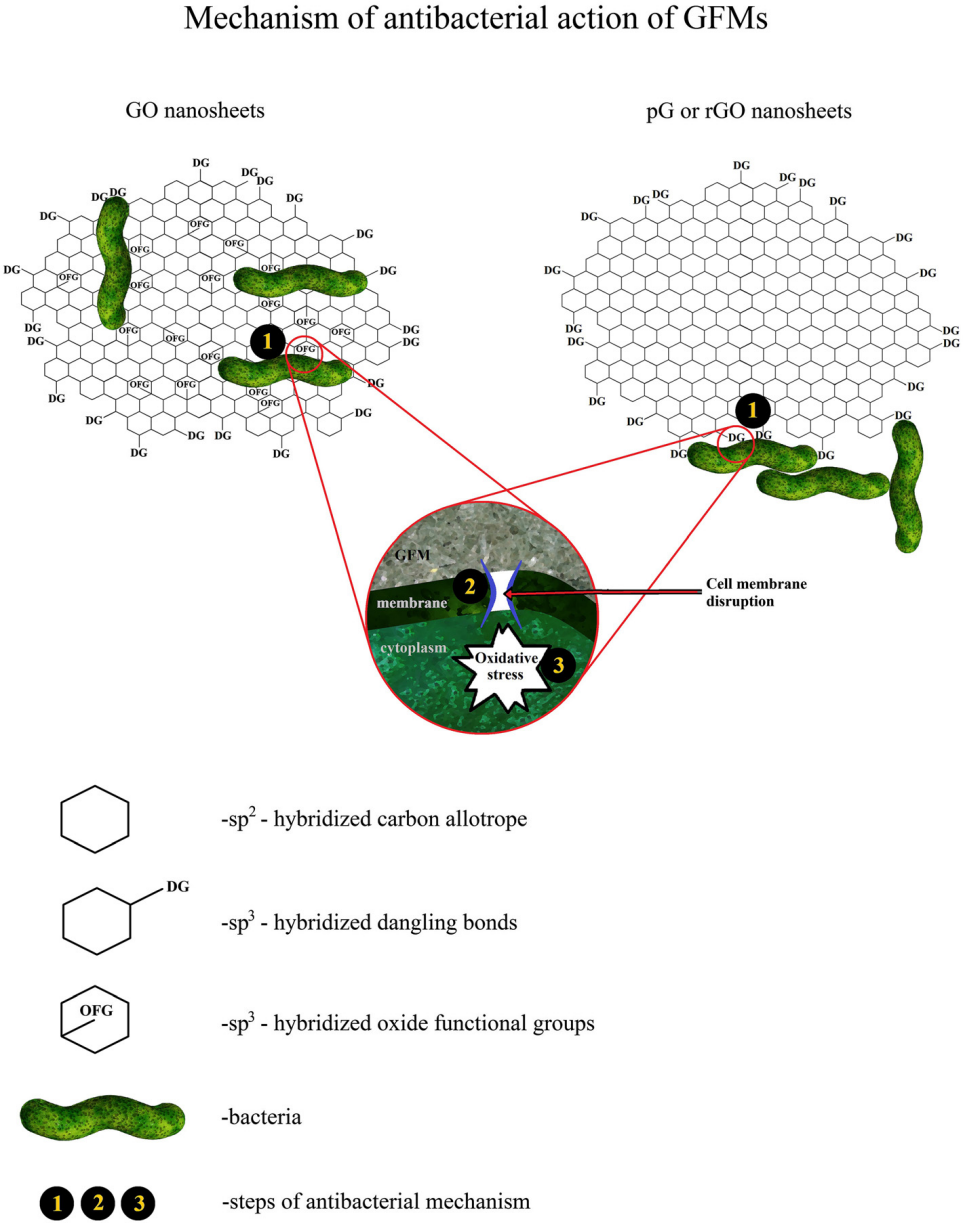
**Three-step antimicrobial mechanism of graphene materials.** 1. Initial bacteria cell deposition on graphene materials. 2. Membrane stress caused by direct contact with sharp edges. 3. Oxidative stress in the bacterial cytoplasm. The key difference between the chosen graphene materials is the bacterial cell deposition place. Individual bacterial cells interact with the sp^3^-hybridized oxidative functional group of the GO surface, while bacterial cells interact with the sharp edges of pG and rGO and form a rope-like structure.

The present results indicate that GFM antibacterial activity causes mechanical damage of bacterial cell membranes by a direct contact of the bacteria with the extremely sharp edges of GFM with sp^3^-hybridized bonds. This mechanical damage can be enhanced by the oxidative stress in bacteria. Based on the present results, we propose a three-step antimicrobial mechanism of GFM outlined in Figure 9. It includes initial cell deposition on GFM (step 1), membrane stress and disruption caused by direct contact with sharp edges and bonds (step 2), and finally stimulated oxidation stress (step 3). The difference in bacteria deposition observed between pG, rGO, and GO sheets in step 1 of the antibacterial mechanism might arise from different surface charges and functional groups of GO and rGO surfaces. Akhavan and Ghaderi [22] measured the efflux of cytoplasm materials of the bacteria after contact with sharp edges of the nanowalls and suggested that it was the effective mechanism of bacterial inactivation. Furthermore, the TEM studies revealed that *E. coli* largely lost cellular integrity after exposition to GO and rGO, with the cell membrane being severely destroyed and the cytoplasm flowing out, which might arise from the effects of either oxidative stress or physical disruption [19].

The current few cytotoxicity studies on graphene materials suggest some similarity between graphene and other carbon nanomaterials. According to the present results, GO is the most efficient and antibacterial substance compared to pG and rGO. Graphene-based nanomaterials can effectively inhibit the growth of *E. coli* bacteria while showing minimal cytotoxicity; GO nanosheets (20 μg/mL) exhibited no cytotoxicity to A549 cells, indicating that GO nanosheets are relatively biocompatible nanomaterials with mild cytotoxicity, but they almost entirely suppressed the growth of *E. coli*, leading to a viability loss up to 98.5%. Furthermore, rGO possessed antibacterial properties that were only slightly lower than those of GO, while their cytotoxicity was significantly higher than GO's [19]. Liu et al. [20] reported that under similar concentration and incubation conditions, GO had higher antibacterial activity than rGO. Moreover, the GO nanowalls reduced by hydrazine were more toxic to the bacteria than the unreduced GO nanowalls [22].

The data generated in this study indicated that the surface of GO effectively attracted and strongly bound bacterial cells, while only the edges of rGO and pG were targeted by bacteria. Despite this, however, both rGO and pG had antibacterial activity. This suggests that surfaces covered with graphene nanolayers, irrespective of their form (pG, GO, rGO), may have bacteria-resistant properties that could be useful for medical applications.

## Conclusions

Independent of the method of production, pG and rGO have similarities in surface chemistry but significantly differ from GO. The characterization of their antibacterial properties greatly reflected this, particularly in relation to their interaction with the tested bacteria. All the GFM had antibacterial properties but to different extents and via varied mechanisms. Bacteria attached to the edges of pG and rGO flakes rather than surrounding them, in contrast to GO, which had attached bacteria, distributed over its surface. It was also demonstrated that high concentrations of all the GFM had detrimental effects on bacterial growth. Of the different GFM, GO was found to have the highest antibacterial activity also at a low concentration.

## Abbreviations

GFM: graphene family materials
GO: graphene oxide
pG: pristine graphene
rGO: reduced graphene oxide
SEM: scanning electron microscope
TEM: transmission electron microscope
